# Development of a Network-Based Signal Detection Tool: The COVID-19 Adversome in the FDA Adverse Event Reporting System

**DOI:** 10.3389/fphar.2021.740707

**Published:** 2021-12-08

**Authors:** Michele Fusaroli, Emanuel Raschi, Milo Gatti, Fabrizio De Ponti, Elisabetta Poluzzi

**Affiliations:** Department of Medical and Surgical Sciences, Alma Mater Studiorum, University of Bologna, Bologna, Italy

**Keywords:** adversome, COVID-19, adverse drug reactions, pharmacovigilance, network, iatrogenic syndromes

## Abstract

**Introduction:** The analysis of pharmacovigilance databases is crucial for the safety profiling of new and repurposed drugs, especially in the COVID-19 era. Traditional pharmacovigilance analyses–based on disproportionality approaches–cannot usually account for the complexity of spontaneous reports often with multiple concomitant drugs and events. We propose a network-based approach on co-reported events to help assessing disproportionalities and to effectively and timely identify disease-, comorbidity- and drug-related syndromes, especially in a rapidly changing low-resources environment such as that of COVID-19.

**Materials and Methods:** Reports on medications administered for COVID-19 were extracted from the FDA Adverse Event Reporting System quarterly data (January–September 2020) and queried for disproportionalities (Reporting Odds Ratio corrected for multiple comparisons). A network (the Adversome) was estimated considering events as nodes and conditional co-reporting as links. Communities of significantly co-reported events were identified. All data and scripts employed are available in a public repository.

**Results:** Among the 7,082 COVID-19 reports extracted, the seven most frequently suspected drugs (remdesivir, hydroxychloroquine, azithromycin, tocilizumab, lopinavir/ritonavir, sarilumab, and ethanol) have shown disproportionalities with 54 events. Of interest, myasthenia gravis with hydroxychloroquine, and cerebrovascular vein thrombosis with azithromycin. Automatic clustering identified 13 communities, including a methanol-related neurotoxicity associated with alcohol-based hand-sanitizers and a long QT/hepatotoxicity cluster associated with azithromycin, hydroxychloroquine and lopinavir-ritonavir interactions.

**Conclusion:** Findings from the Adversome detect plausible new signals and iatrogenic syndromes. Our network approach complements traditional pharmacovigilance analyses, and may represent a more effective signal detection technique to guide clinical recommendations by regulators and specific follow-up confirmatory studies.

## Introduction

The COVID-19 pandemic forced the medical community to confront a scenario of extreme uncertainty, variegate use of unapproved drugs (candidates for drug repurposing), and rolling reviews of experimental therapeutics. This led to an unprecedented request for early and timely signal detection of adverse reactions: a call to action for pharmacovigilance studies, which provide a quick, inexpensive, and relatively easy approach to real-time monitoring of emerging safety issues through the analysis of spontaneous reporting systems. Early in the COVID-19 outbreak, pharmacovigilance studies attempted to characterize the safety profile of repurposed agents using data gathered outside the COVID-19 context, as an aid to prioritize monitoring and raise clinicians’ awareness in patients with COVID-19 disease. For instance, tocilizumab and hydroxychloroquine, traditionally employed in rheumatoid arthritis, were widely–albeit temporarily for hydroxychloroquine–repurposed for COVID-19. Pharmacovigilance studies indicated that hepatic and cardiovascular events should be monitored in patients administered with these drugs (Gatti et al., 2021; Goldman et al., 2021). As the pandemic progressed, pharmacovigilance studies, using data generated in the COVID-19 setting, have started to accrue. These studies uniquely provide real-world evidence on the toxicity spectrum and reporting pattern of COVID-19 drugs, and give the chance to seriously discuss the methodological issues and challenges faced by pharmacovigilance studies in COVID-19 (Garcia et al., 2020; Montastruc et al., 2020; Zekarias et al., 2020).

Pharmacosurveillance studies are a fundamental tool for drug safety, complementary to clinical trials because they address the complexity of real-world data, such as extreme ages, polytherapy, comorbidity, misuse, and medical errors. In particular, pharmacovigilance, through the so-called disproportionality approach, has attracted considerable interest by clinicians for its ability to timely and early assess emerging toxicities in recently approved drugs in different therapeutic domains (Raschi et al., 2020a). The strengths and limitations of these analyses are still being debated: e.g., the actual correlation of disproportionality findings with relative risk (Khouri et al., 2021a) and the considerable variability in results emerging from yet to settle key methodological issues (Khouri et al., 2021b). In the recent past, methodological efforts have mainly focused on the performance of disproportionality analyses: how to choose amongst the several spontaneous reporting systems (Vogel et al., 2020), approaches and relevant thresholds (Candore et al., 2015), database type and size (Caster et al., 2020), as well as how to identify and account for reporting biases (Arnaud et al., 2016). However, disproportionality analyses, comparing the reports of specific drugs and adverse events as cases and all other reports as non-cases, have also been criticized since they do not consider other clinical information (concomitant drugs and events, indications, age, gender, weight). Sensitivity/subgroup analyses are a way to include some of these aspects in the disproportionality study, but new specific tools are needed to properly delve into this complexity.

Network Medicine (Barabási et al., 2011) may be useful for investigating the complex interdependencies within spontaneous reporting data information. Other health-related fields are benefiting from network science: intracellular networks [Interactome (Venkatesan et al., 2009)], genes-diseases association [Diseasome (Goh et al., 2007)], comorbidities of psychopathological symptoms (Borsboom et al., 2011), and drug-target interaction (Cheng et al., 2012). FAERS data described in the indication field have also been used to build a disease comorbidity network to investigate gene sharing for drug repurposing (Zheng and Xu, 2018). Furthermore, a specific net of adverse events has been proposed to identify terms co-reported with suicide as possible predictors (Nazir et al., 2014). Amongst the different methods to identify network structures from data, the Ising model is a pairwise Markov random field able to represent conditional dependencies between binary variables, from electromagnetic particle interactions to causal networks of psychopathological signs and symptoms (van Borkulo et al., 2014). It is therefore particularly promising for the evaluation of partial correlations among adverse events in spontaneous reports, being capable of excluding spurious associations due to confounders (Epskamp et al., 2018).

This study proposes a new approach to signal detection that explicitly relies on the multiple suspected Adverse Drug Reactions (ADRs) often reported: the Adversome. A network approach could support an effective visualization of co-reporting, to interpret signals of disproportionate reporting (SDRs) and highlight communities of significantly co-reported events. The identification of commmunities could help distinguish the underlying COVID-19 contribution on adverse events/syndromes from common comorbidities and the actual role of suspected drugs, and may manifest itself as extremely useful for the early detection and prioritization of signals in a rapidly changing low-resources envirnonment such as that of COVID-19.

## Methods

### Data Source

The FDA Adverse Event Reporting System (FAERS) is a freely available spontaneous reporting system that collects worldwide reports of suspected ADRs. Its data can be accessed both on a public dashboard (FDA, 2021a), for quick but superficial surveys, and as raw quarterly data (FDA, 2021b) (Quarterly Data), which allows for thorough deduplication and in-depth customized analysis. Both reaction and indication fields are coded according to the Medical Dictionary for Regulatory Activities (MedDRA) terminology. MedDRA is a hierarchical lexicon with Preferred Terms (PTs) for standardized pharmacovigilance practice. PTs are gathered in High-Level Terms (HLTs), which are grouped in High-Level Group Terms (HLGTs), included in specific System Organ Classes (SOCs). Although a PT can be included in many SOCs, one of them is defined as the primary one.

### Exposure of Interest

We extracted and pre-processed–for both deduplication and standardization–FAERS quarterly data and selected reports with the date of occurrence–or, if missing, of the first report to the FDA–from January to September 2020. Although ADRs are coded in the FAERS as MedDRA PTs, to account for dictionary redundancy and the small size of the population of interest (and display fewer larger nodes in the network), we grouped them into their primary HLTs. We have not considered HLTs included in the following, non-specific, SOCs: *Injury, poisoning and procedural complications*; *Social circumstances*; *Product issues*; *Surgical and medical procedures*; *Investigations*; *General disorders and administration site conditions*. To select reports regarding COVID-19 we searched the narrow Standardized MedDRA Query (SMQ) for Sars-Cov-2 (see Supplementary Table S1) in the indication field, an algorithm already developed for pharmacovigilance purposes in COVID-19 (Zekarias et al., 2020). The broad SMQ has not been adopted because it includes also terms concerning negative test results. The descriptive analysis of COVID-19 reports was performed with non-COVID-19 reports of the same period as a reference.

### Disproportionality Analysis

Reporting Odds Ratio (ROR) was used as a measure of disproportional reporting with drugs mentioned as suspected in more than 100 reports. To reduce the risk of false detection of disease-related conditions, the disproportionality analysis was performed within COVID-19 reports. According to good signal detection practice (Reynolds et al., 2016), we calculated the ROR only in the presence of at least 3 cases, and we considered it statistically significant when its entire 95% confidence interval (CI) was greater than 1. Furthermore, to control type 1 error inflation due to multiple testing (i.e., false alarms), the *p*-value of the Fisher Exact test was adjusted using Bonferroni correction: the 0.05 α threshold for *p*-value significance was divided by the number of tests performed. An SDR was identified only when both 95% CI and corrected *p*-value were significant. A heat map was designed to visualize the disproportionalities grouped by SOC.

### Network Analysis: The Adversome

For the Adversome, ADRs with at least 10 cases in COVID-19 were selected as nodes and their co-reporting relationships as links. The reaction field was converted to binary data (i.e., presence 1 or absence 0 of each event in each report), and the weight of the links was calculated as the partial correlation between each pair of variables (nodes) conditioning on all other variables in the network. The LASSO (least shrinkage and selection operator) was used to remove small-amplitude links, thus obtaining a low rate of false positives at the expense of losing weak true associations (Epskamp et al., 2018). Finally, a multi-level unsupervised community-detection algorithm (Louvain) was used to group commonly co-reported ADRs, thus defining syndrome-like frameworks. Assortativity (i.e., the tendency of nodes sharing an attribute to connect, ranging from −1 to 1) was calculated for degree (i.e., number of links), disproportionality, and primary SOC to investigate co-reporting mechanisms.

### Statistical Tools

Pre-processing, statistical analysis, and plotting were performed using the “R” software (v. 4.02) and the following free of charge R packages: *tidyverse*, *data.table*, *NetworkToolbox*, *visNetwork*, *igraph*, *qgraph*, *IsingFit*. Data and scripts developed are available in a public repository (Fusaroli et al., 2021).

## Results

### Descriptive Analysis

7,082 COVID-19 reports were extracted, comprising 387 different HLTs (132 with less than three reports), and 998 different active ingredients (336 with less than three reports). In Table 1 we listed the number of COVID-19 reports related to the 10 most frequent HLTs, the 10 most frequent active ingredients, and the 10 most frequent primary suspect active ingredients. The most-reported HLTs, in addition to coronavirus infection, were the following: renal failure and impairment (10% of reports); hepatocellular damage and hepatitis NEC–not elsewhere classified–(5%); ventricular arrhythmias and cardiac arrest (5%); respiratory failure (5%). The most-reported drugs were remdesivir (47% of reports), hydroxychloroquine (38%), and azithromycin (31%). Drugs reported as primary suspects in more than 100 reports were remdesivir (39%), hydroxychloroquine (13%), azithromycin (10%), tocilizumab (8%), lopinavir (5%, in combination with ritonavir), sarilumab (2%), and ethanol (2%). These seven drugs were then used in the disproportionality analysis.

**TABLE 1:** Main drugs and events. Ranking of reactions (HLTs, High Level Terms), Active Ingredients, and Primary Suspect (PS) Active Ingredients in COVID-19 patients.

Rank	Events (HLTs)	N° reports (%)	Active ingredients	N° reports (%)	PS active ingredients	N° reports (%)
1	Renal failure and impairment	730 (10.3%)	Remdesivir	3,342 (47.2%)	Remdesivir	2,744 (38.8%)
2	Coronavirus infections	534 (7.5%)	Hydroxy chloroquine	2,717 (38.4%)	Hydroxy chloroquine	939 (13.3%)
3	Hepatocellular damage and hepatitis NEC	388 (5.4%)	Azithromycin	2,162 (30.5%)	Azithromycin	702 (9.9%)
4	Ventricular arrhythmias and cardiac arrest	365 (5.2%)	Enoxaparin	1,420 (20.0%)	Tocilizumab	584 (8.3%)
5	Respiratory failures (excluded neonatal)	358 (5.1%)	Tocilizumab	1,260 (17.8%)	Lopinavir	364 (5.1%)
6	Rate and rhythm disorders NEC	277 (3.9%)	Ceftriaxone	1,193 (16.9%)	Ritonavir	335 (4.7%)
7	Breathing abnormalities	230 (3.3%)	Ritonavir	1,186 (16.8%)	Sarilumab	151 (2.1%)
8	Sepsis, bacteremia, viraemia, and fungaemia NEC	226 (3.2%)	Lopinavir	1,160 (16.4%)	Ethanol	123 (1.7%)
9	Pulmonary oedemas	192 (2.7%)	Paracetamol	914 (12.9%)	Methyl prednisolone	100 (1.4%)
10	Nausea and vomiting symptoms	183 (2.6%)	Dexamethasone	849 (12.0%)	Oseltamivir	91 (1.2%)

When COVID-19 and non-COVID-19 reports were compared (Table 2), a higher proportion of reports was found in men (63 *vs.* 39%), elderly (41 *vs.* 35%), Spain and Italy (respectively 10 *vs.* 1%, and 7 *vs.* 1%). COVID-19 cases were submitted more by pharmacists (42 *vs.* 7%), and with a reporting peak in June and July. Death and life-threatening outcomes were more frequently reported in the COVID-19 group (respectively 24 *vs.* 9%, and 7 *vs.* 2%).

**TABLE 2:** Descriptive analyses. Main characteristics of reports in COVID-19 and non-COVID-19 patients (1q-3q 2020).

		COVID-19	Non-COVID-19
Sex	Female	2318 (37.1%)	334645 (60.7%)
	Male	3935 (62.9%)	217061 (39.3%)
	Missing	829	85114
Country	United States	4020 (56.8%)	454568 (71.4%)
	Spain	714 (10.1%)	5007 (0.7%)
	France	587 (8.3%)	13848 (2.2%)
	Italy	468 (6.6%)	7745 (1.2%)
	Switzerland	150 (2.1%)	7776 (1.2%)
	Other/Missing	1143	147876
Occupation	Pharmacist	2822 (42.1%)	44190 (7.2%)
	Medical doctor	1747 (26.1%)	119344 (19.4%)
	Healthcare practitioner	1592 (23.8%)	145116 (23.6%)
	Consumer	536 (8.0%)	298649 (48.6%)
	Lawyer	0 (0.0%)	7121 (1.2%)
	Missing	385	22400
Report code	Expedited	4306 (60.8%)	314980 (49.5%)
	Direct	2684 (37.9%)	46074 (7.2%)
	Periodic	92 (1.3%)	275766 (43.3%)
Age group	Elderly	2519 (41.2%)	124936 (35.4%)
	Adult	3467 (56.7%)	202124 (57.3%)
	Teenager	61 (1.0%)	13740 (3.9%)
	Child	41 (0.7%)	7307 (2.1%)
	Infant	21 (0.3%)	3671 (1.0%)
	Newborn	4 (0.1%)	695 (0.2%)
	Missing	969	284347
First report	Jan	0 (0.0%)	60556 (9.5%)
	Feb	4 (0.1%)	66473 (10.4%)
	Mar	51 (0.7%)	72611 (11.4%)
	Apr	420 (5.9%)	71408 (11.2%)
	May	1219 (17.2%)	69718 (10.9%)
	Jun	1647 (23.3%)	75001 (11.8%)
	Jul	1483 (20.9%)	77772 (12.2%)
	Aug	1177 (16.6%)	69309 (10.9%)
	Sep	1081 (15.3%)	73972 (11.6%)
Outcome	Death	1690 (23.9%)	54071 (8.5%)
	Life-threatening	470 (6.6%)	12785 (2.0%)
	Disability	49 (0.8%)	6828 (1.1%)
	Required-intervention	38 (0.6%)	371 (0.1%)
	Hospitalization	1176 (19.2%)	108805 (17.1%)
	Congenital anomaly	3 (0.0%)	1253 (0.2%)
	Other serious	2696 (44.0%)	166439 (26.1%)
	No seriousness specified	960 (13.6%)	286268 (45.0%)

### Disproportionality Analysis

**FIGURE 1:**
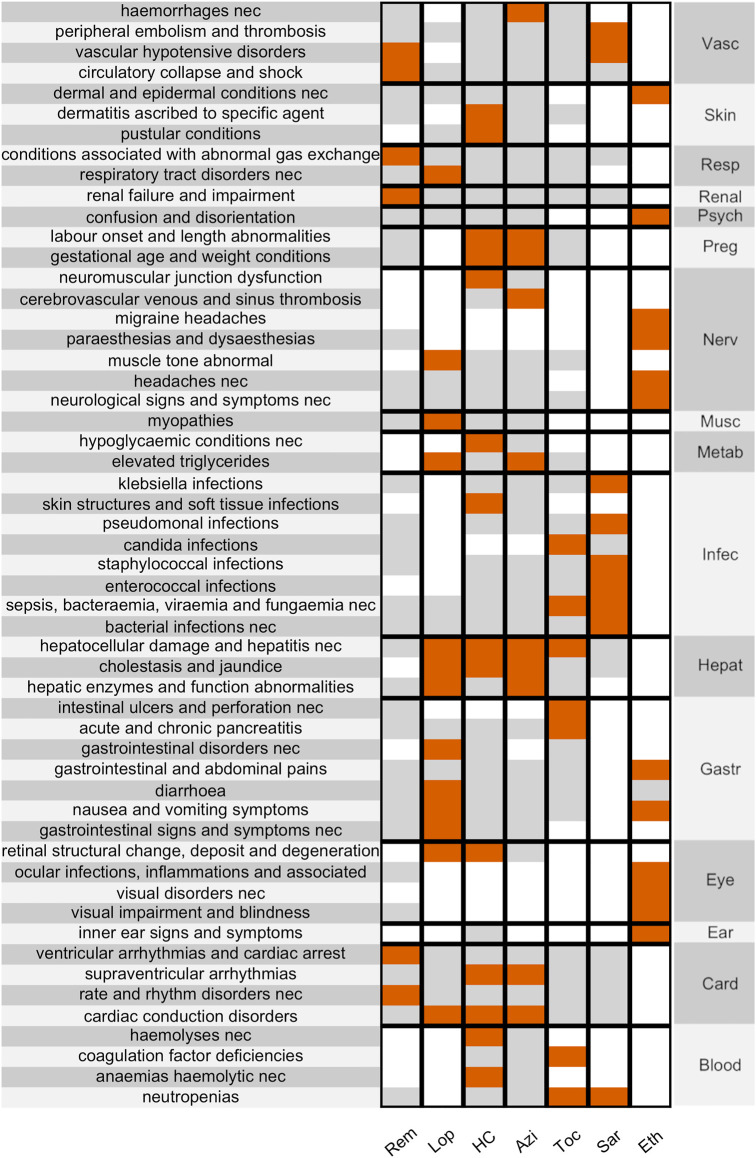
Disproportionality Heat Map. showing significant disproportionalities (after the Bonferroni correction) between HLTs (clustered by SOC, on the **right**) and the main suspected drugs in COVID-19 patients. Associations are colour-coded (white when not calculated, grey when not significant, red when significant). SOCs: Vascular, Skin, Respiratory, Renal, Psychiatric, Pregnancy, Nervous, Muscular, Metabolic, Infective, Hepatic, Gastrointestinal, Eye, Ear, Cardiac, Blood. Drugs: remdesivir, lopinavir, hydroxychloroquine, azithromycin, tocilizumab, sarilumab, ethanol.

Focusing on the seven drugs, 54 out of 387 HLTs resulted in disproportionality for at least one drug (148 before the Bonferroni correction—619 analyses performed, *α* = 0.000080775–). The significant disproportionalities were shown, gathered by SOC, in the heat map in Figure 1 (before correction in the Supplementary Figure S1, with RORs made available in the Supplementary Table S2). Major findings concerned the following: lopinavir/ritonavir, hydroxychloroquine, and azithromycin with cardiac and liver conditions; hydroxychloroquine with skin conditions; lopinavir with gastrointestinal conditions; hydroxychloroquine and azithromycin with pregnancy conditions; sarilumab with blood and infection conditions; ethanol with ocular, nervous, and psychiatric conditions; remdesivir with cardiac, hypotensive, and renal conditions.

### Network Analysis: The Adversome

**FIGURE 2:**
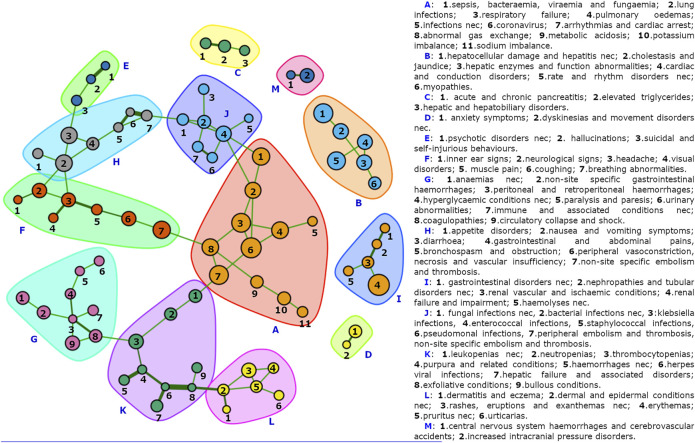
The COVID-19 Adversome. Showing only connected nodes, automatically grouped via a multi-level detection algorithm. Individual clusters are available in the supplementary material, and the full interactive network is available in the OSF public repository (Fusaroli et al., 2021).

A subnetwork of the Adversome with only positively connected nodes and identified communities is represented in Figure 2, and binary data, code and interactive version of the network are also made available on a OSF public repository (Fusaroli et al., 2021). The undirected weighted network of reactions includes 136 nodes and 87 undirected links (83 green/positive, four red/negative). Only 77 nodes have at least one link. There is a weak assortativity for nodes with the same degree (0.0687), and a stronger one for nodes sharing disproportionality (0.3726) or the primary SOC (0.4388). The multi-level community-detection algorithm identifies 13 clusters, which we referred to using letters from A to M. Cluster A includes coronavirus and respiratory conditions and represents the core of the network. It is connected via K to L and G, and via F and J to H and then to E. 5 clusters (B, C, D, I, M) are disconnected from the others. Looking at the interactive network, B and I are also mutually exclusive (red link) to coronavirus and respiratory failure. Disproportionalities with clusters (visualized in the interactive network stored in the available repository, where nodes can be specifically selected by significant ROR) occur for azithromycin and hydroxychloroquine with B nodes, ethanol with H and F, lopinavir/ritonavir with B and H, remdesivir with respiratory conditions, and sarilumab with J.

## Discussion

### Where Do We Stand

New drugs, for which safety was tested only as a secondary outcome in small numbers and ideal conditions-clinical trials, are introduced continuously in the real world, and require a strict monitoring by pharmacovigilance. COVID-19 pandemic, with its rapidly changing environment and limited resources, and with the repurposing of already marketed agents, has shown an even stronger need for pharmacovigilance and its signal detection.

However, current methods (e.g., single disproportionality analyses) struggle with two issues: 1) the richness and complexity of cases, often containing multiple drugs and adverse events; and 2) the presence of multiple important biases confounding the inference of associations as reaction.

In order to deal with these issues we developed the Adversome, a new approach that complements traditional signal detection. The Adversome explicitly takes into account the co-reporting of multiple suspected Adverse Drug Reactions (ADRs) and helps to include this information in the clinical assessment of statistical signals, as a way to better detect and correct for multiple biases.

We will now discuss the findings achieved by applying the Adversome to the specific issue of repurposed drugs in the context of the COVID-19 pandemic. In particular, after describing the demographics of the population investigated and the old and new signals detected through the disproportionality analysis, we will discuss the contribution of the Adversome to signal assessment. We will conclude discussing the limitations, strengths, and perspectives of the study.

### Epidemiology

Reports with COVID-19 drugs show high complexity, reporting the same distribution of other adverse events [2 (1–3) PTs per record], but twice as many number of drugs administered [4 (2–8) *vs.* 2 (1–4) drugs per record]. Their characteristics are in line with the epidemiology of COVID-19, with males and the elderly being more represented, more frequent deaths, and high contributions from countries heavily affected by the first wave such as Italy and Spain. Interestingly, some peculiar submission characteristics emerged: 38% reports were sent via MedWatch (*vs.* 7%), and 42% by pharmacists (*vs.* 7%).

Adverse events reported in the reaction field are both common suspected ADRs (renal impairment, hepatocellular damage, arrhythmias) and disease-related terms (e.g., coronavirus infection, respiratory failure, breath abnormalities, viremia, pulmonary edema, gastrointestinal symptoms). Medications reported in the drug field are heterogeneous and encompass antivirals, antibiotics, monoclonal antibodies, glucocorticoids, supplements (e.g., zinc, ascorbic acid, vitamin D), heparins, acetaminophen, NSAIDs, and drugs used for chronic conditions (e.g., insulin and statins). Ethanol, as an active ingredient of hand sanitizers, is not one of the most reported substances, but when present it is almost always the primary suspect (123/128 cases, *vs.* 702/2,162 cases of azithromycin). This could be due to the very common use of sanitizers, which was likely not reported unless strongly suspected.

### Old and New Signals

The SDRs that have emerged must be interpreted through clinical and pharmacological knowledge because even a strong association does not always mean causality. To discriminate between disease-related and drug-induced conditions, we performed the disproportionality analysis within the population of COVID-19 reports. By doing so, cases and controls share disease-related conditions, and therefore the ROR for these events should be regarded as non significant. Nonetheless, this may not apply if there is a differential use of drugs in the presence of the event investigated. For example, coagulation factor deficiencies with tocilizumab, and peripheral embolism and thrombosis with sarilumab, are presumably related to the use of these anti-IL6 to treat the pro-coagulative cytokine storm, characteristic of severe COVID-19 (Kichloo et al., 2020; Xu et al., 2020), suggesting an indication bias. Nonetheless, it should also be considered that some evidence of thrombophilic side effects of tocilizumab is starting to accumulate (Chan et al., 2021).

It should also be noted that known manifestations of a disease can also be induced, through other mechanisms, by the drugs administered to treat it (e.g., gastrointestinal conditions with lopinavir/ritonavir), and that an amplified masking bias may occur, due to the small size of the population of interest, between drugs associated with the same event.

The SDRs concerning already detected associations are the following: hemolytic anemia and hemolysis with hydroxychloroquine (Beauverd et al., 2020); Drug-Induced Liver Injury (DILI) (Gatti et al., 2021), pancreatitis and intestinal ulcer with tocilizumab; hypoglycemia (Dirim et al., 2020) and cutaneous adverse events with hydroxychloroquine (Sharma et al., 2020); enterotoxicity and myopathy with lopinavir/ritonavir; infections with sarilumab and tocilizumab; conduction disorders (mostly long QT syndrome) and DILI with lopinavir/ritonavir, hydroxychloroquine, and azithromycin; retinopathy with hydroxychloroquine and lopinavir/ritonavir; hypertriglyceridemia with lopinavir/ritonavir and azithromycin. Already emerging disproportionalities concern neuromuscular joint dysfunction (entirely myasthenia gravis) with hydroxychloroquine (Jallouli et al., 2012; Elavarasi and Goyal, 2020); bradycardia (Touafchia et al., 2021), hypotension, and kidney injury (Charan et al., 2021) with remdesivir. Regarding remdesivir-induced bradycardia, it should be taken into account that a channelling bias could apply, at least partially, since an important decrease in heart rate is a known physiological response to worsening hypoxemia (Higgs et al., 2019) (also disproportionally associated with remdesivir).

An active ingredient of particular interest is ethanol, contained in alcohol-based sanitizers. It is associated with reports of nausea and vomiting, abdominal pain, headache, paresthesia, dermatitis, vertigo, confusion, and visual impairments. Notably, an FDA warning of June 2020, constantly updated (Research C for DE and. FDA updates on hand sanitizers consumers should not use, 2021), specifically addressed adverse events induced by hand-sanitizers and has prompted the voluntary withdrawal of multiple ethanol-based sanitizers due to traces of methanol, including Blumen (32% of our reports), and Next products (8% of our reports).

Gestational conditions associated with azithromycin and hydroxychloroquine (almost entirely premature and low weight baby) may require further analyses but may also be related to a channelling bias (these drugs could have simply been the choice in women because they can be administered at home and have a long history of use in pregnancy without special warnings). Another channelling bias could explain the association of respiratory conditions with remdesivir and lopinavir/ritonavir, used primarily in more symptomatic COVID-19. Another association that requires further consideration is that of cerebrovascular venous and sinus thrombosis with azithromycin, of which some cases have been recorded in clinical practice (Cavalcanti et al., 2020).

Finally, it should be noted that the correction for multiple comparisons (Fadini et al., 2018), is not commonly adopted in signal detection because it is too conservative and therefore not adequate in a context where a more speculative detection of adverse events using early post-marketing data is required. Therefore, we cannot rule out that associations thus rejected as false alarms could instead be considered of interest in targeted studies, which may evaluate them using other tools to support the existence of causation, such as the Bradford-Hill criteria (Raschi et al., 2020b). One example is the association of hydroxychloroquine with psychiatric conditions such as suicidal and self-injurious behaviors and hallucinations with hydroxychloroquine. This association has indeed been detected, in COVID-19 patients in the WHO spontaneous reporting system (Garcia et al., 2020), although there is also contrary evidence pointing to the psychiatric safety of hydroxychloroquine in rheumatoid arthritis (Lane et al., 2020).

### Iatrogenic Syndromes

Our COVID-19 Adversome enables the visualization of significant co-reporting relationships and identifies groups of events that maximize within-group and minimize between-groups co-reporting. By segregating groups of signs and symptoms that commonly occur together after administration of drug therapy, it identifies potential iatrogenic syndromes. Furthermore, an SDR concerning a drug that is disproportionally associated with a neighborhood of nodes may be considered as more reliable: i.e., there is indeed a cluster of symptoms that commonly co-occur together and with the drug.

Cluster A (coronavirus, and respiratory and metabolic conditions) represents the heart of the network, presumably attributable to the disease. In fact, its nodes are not disproportionally associated, within the COVID-19 population, with any of the drugs investigated (apart from the already discussed remdesivir-related bradycardia and the sarilumab-related sepsis, bacteremia, viremia and fungemia NEC).

It is linked through sepsis to the infection-related cluster J. The association of all J nodes (apart from fungal infections NEC) with sarilumab supports its already suspected role in increasing the susceptibility to infections, providing much stronger evidence than that of a single significant ROR, or even of multiple individual significant RORs.

**FIGURE 3:**
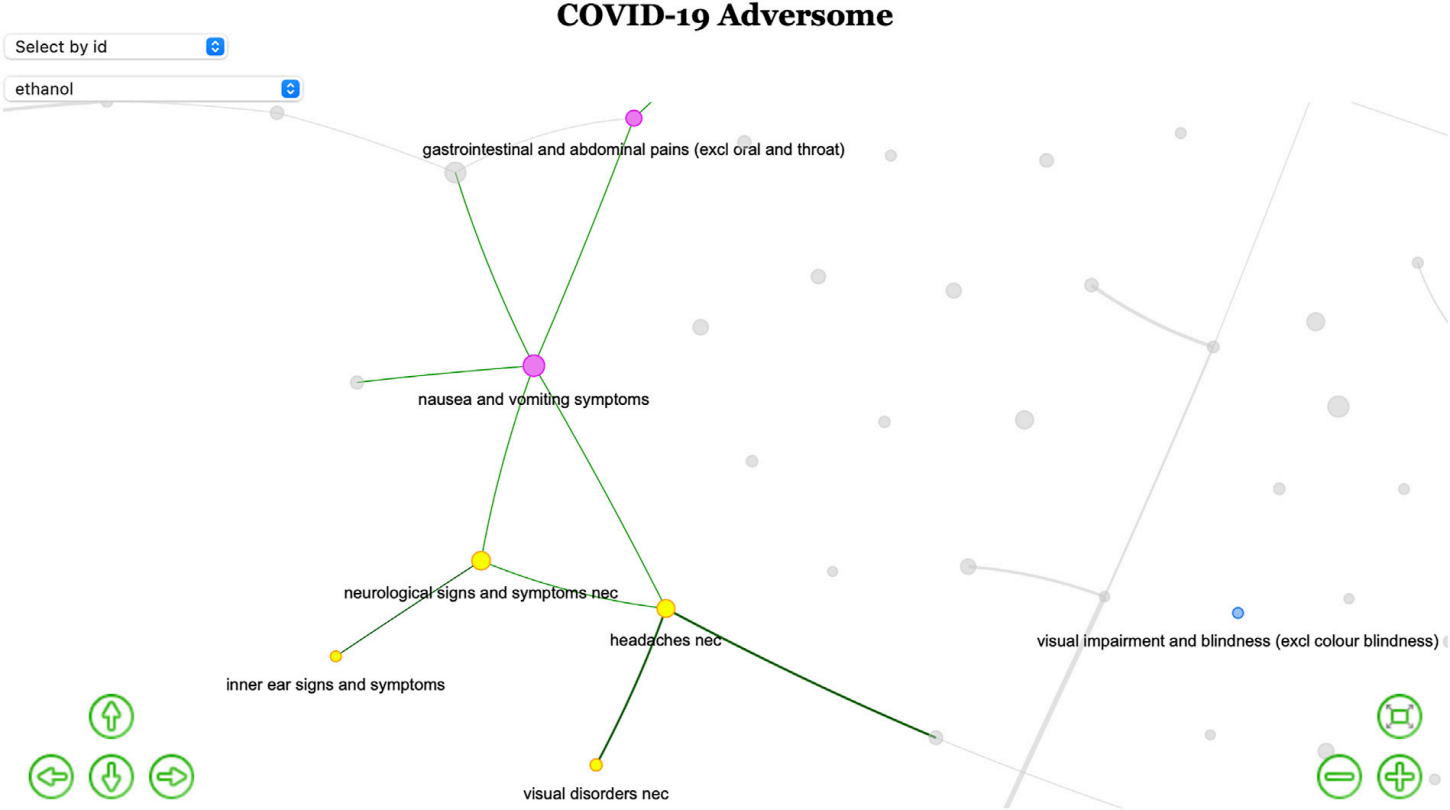
Ethanol-based hand-sanitizers toxicity. Screenshot of part of the interactive network (available in the OSF public repository Fusaroli et al., 2021) highlighting only nodes disproportionally associated with ethanol. The existence of a iatrogenic syndrome is here suggested by the colored subgraph of co-reported events.

Nausea and vomiting and abdominal pain (cluster H) are linked to cluster F (neurological signs, vertigo, and visual impairment), with which they share disproportionality for ethanol. The Adversome turned out to be extremely useful in identifying and visualizing this methanol-related toxicity that led to the voluntary withdrawal of many alcohol-based hand-sanitizers during the pandemic (Figure 3).

Cluster B is of great interest and would require more specific studies, as it groups hepatocellular damage, long QT syndrome, and myopathy. This pattern of co-reported events could be related to QT interval prolongation in severe liver damage (Arya et al., 2020), but also to reduced clearance and frequent combination of three drugs disproportionally associated with the cluster and known to induce long-QT and liver injury (Roy and Mukhopadhyay, 2020): of the 3,487 reports with at least one of them, 1,042 (29.9%) reported both hydroxychloroquine and azithromycin, 361 (10.4%) both lopinavir/ritonavir and hydroxychloroquine, 64 (1.8%) both lopinavir/ritonavir and azithromycin, 315 all three drugs (9.0%). These clusters are thus presumably attributable to drugs.

Gastrointestinal conditions (cluster H) are linked through diarrhea to hallucinations, suicide, and psychotic symptoms (cluster E), associated with hydroxychloroquine only before Bonferroni correction. Cluster C covers pancreatic conditions (associated with tocilizumab only before Bonferroni), D anxiety, I kidney injury, and M stroke. These clusters are more difficult to refer to disease or drug.

Clusters G and K concern pancytopenia, linked through herpes simplex to dermal conditions (cluster L). Within coagulation disorders, peritoneal and retroperitoneal hemorrhages are connected to a chain of symptoms that could be related to diabetes (hyperglycaemic conditions, paralysis and paresis, urinary abnormalities), thus possibly identifying a comorbidity sub-cluster.

Interestingly, for HLTs, being part of the same System Organ Class or being disproportionally reported with the same list of drugs lead to a higher likelihood of being co-reported (positive assortativity). The positive assortativity of SOC can be partly explained by MedDRA redundancy and the fact that symptoms of the same organic dysfunction can be reported separately (e.g., urticarias and pruritus, hemorrhages and purpura, pulmonary edema and respiratory failure), but at the same time it helps to identify organ-specific toxicities. On the other hand, positive assortativity due to disproportionality partly indicates a shared etiology (e.g., hepatic damage and cardiac conduction disorders, induced by azithromycin, hydroxychloroquine, and lopinavir/ritonavir), and a differential use in specific conditions (e.g., tocilizumab and sarilumab used to treat pro-coagulative cytokine storm can cause immunodeficiency, potentially explaining the relationship between thromboembolic events and infections). The following links of interest could support further investigations: diarrhea and hallucinations, staphylococcal infection and peripheral embolism and thrombosis (Liesenborghs et al., 2018), hyperglycaemic conditions, and peritoneal and retroperitoneal hemorrhages. The fact that hypoxia (cluster A) and bradycardia (cluster B), both disproportionally reported with remdesivir, do not have significant co-reporting supports the causal role of remdesivir in inducing bradycardia (*vs.* the hypothesis of a role of hypoxia).

### Limitations and Strengths

The current study presents a major limitation due to the hypothesis-generating nature of pharmacovigilance: the true incidence remains unknown because of the wide range of factors influencing voluntary reporting and the lack of accurate number of exposed patients. Therefore, causality in the association between drugs and adverse events cannot be inferred, and a higher ROR does not equate to a higher individual patient risk. Indeed, we do not claim to assess risk. Further, our network approach attempted to account for the major common biases (e.g., we pre-processed data for duplicates removal, and selected only adverse events reported within the COVID-19 setting).

The main strengths of our study are the opportunity to easily visualize complex intertwined data and the highly conservative approach: along with good signal detection practice we performed the Bonferroni correction for multiple comparisons, strengthening the specificity of our disproportionality analysis to reduce false positives (at the cost of losing some sensitivity). Furthermore, the Ising estimation of the network has already been proven methodologically solid in the study of the relations between symptoms (even if not on spontaneous reporting data), and its Lasso procedure for filtering out spurious associations further improves the specificity of our method.

## Conclusion and Perspectives

Our study details and showcases a new network approach (Adversome) to complement traditional descriptive and disproportionality analyses in order to more effectively study the co-signalling of multiple ADRs. Through the Adversome we provide an overview of the possible ADRs that occurred in the first 9 months of 2020 in the context of managing COVID-19. Together with traditional descriptive and disproportionality analyses, we also propose a new network approach to study the co-signaling of multiple ADRs: the Adversome.

Disproportionality analyses confirmed known ADRs, supported emerging ones (hydroxychloroquine-induced myasthenia gravis and remdesivir ADRs), and documented a systemic sanitizers-induced syndrome that led to FDA warnings and withdrawals during the pandemic. Unexpected new SDRs, which need further studies to correct expected bias, concern premature infants with azithromycin and hydroxychloroquine, cerebrovascular vein thrombosis with azithromycin, and paradoxical thrombophilia induced by tocilizumab. Inspection of the Adversome enabled the detection of the following strong co-reporting relationships: long QT-syndrome, liver injury and myopathy; diarrhea and hallucinations; hyperglycemia and retroperitoneal and peritoneal hemorrhages; respiratory and metabolic conditions and COVID-19 infection.

Our network approach, complementary to traditional pharmacovigilance analyses, is both consistent with existing evidence and identifies new signals and plausible iatrogenic syndromes. Even if it still needs validation through multiple network studies in different pharmacovigilance contexts, the Adversome may represent a useful technique for more effective signal detection by regulators and scientists In this regard, combining a network and a disproportionality approach with case-by-case assessment could represent an integrated approach to accurately differentiate drug- *vs.* disease-related contribution, supporting a safer use of medications.

Further applications could also be the comparison of networks of different populations (age, gender, indication, country) or the analysis of dynamic signaling networks over time (to study the effect of regulatory warning and emerging susceptibilities). The complex information inherent in polytherapy (drug field) and comorbidity (indication field) is itself suitable for representation on a network.

## Data Availability

The original contributions presented in the study are included in the article/Supplementary Material, further inquiries can be directed to the corresponding author.
