# Supplementary material for: Development of a Network-Based Signal Detection Tool: The COVID-19 Adversome in the FDA Adverse Event Reporting System
Source: Front Pharmacol. 2021 Dec 8;12:740707. doi: 10.3389/fphar.2021.740707 (PMC8694570; doi:10.3389/fphar.2021.740707)
Supplement: Supplementary file 1 [file DataSheet1.zip › Supplementary material/Data Sheet 1.docx]

Supplementary Material

# Table S1. COVID-19 narrow SMQ. Terms searched in the indication field to select COVID-19 reports.

| sars-cov-2 test positive |
| --- |
| covid-19 |
| suspected covid-19 |
| exposure to sars-cov-2 |
| covid-19 pneumonia |
| asymptomatic covid-19 |
| occupational exposure to sars-cov-2 |
| covid-19 treatment |
| coronavirus test positive |
| coronavirus infection |
| covid-19 immunisation |
| covid-19 prophylaxis |
| exposure to sars-cov-2 |
| sars-cov-2 antibody test positive |
| sars-cov-2 carrier |
| sars-cov-2 sepsis |
| sars-cov-2 test false negative |
| sars-cov-2 viraemia |
| multisystem inflammatory syndrome in children |


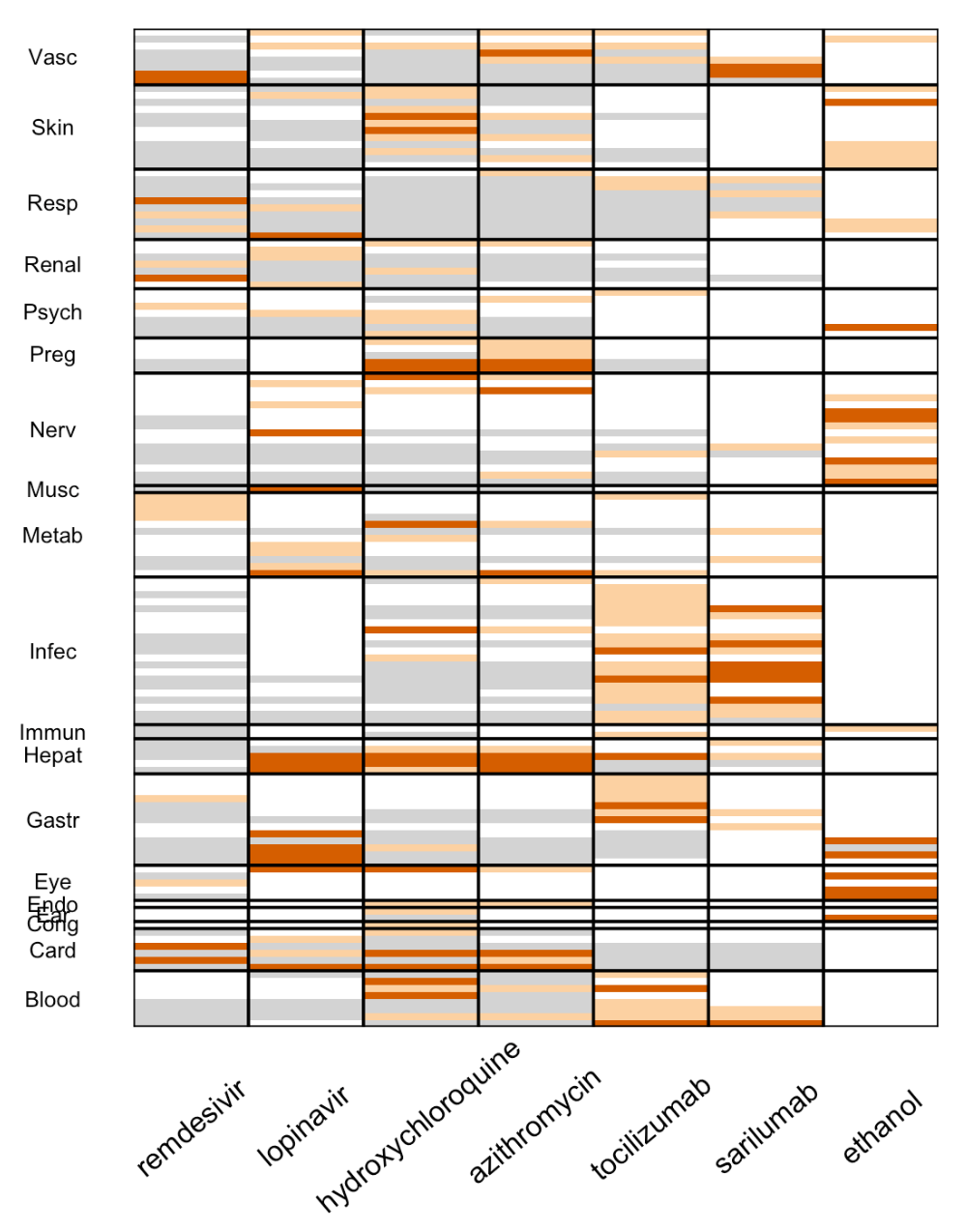


**Figure S1. Speculative Disproportionality heat map**, showing disproportionalities between HLTs (clustered by SOC) and the main suspected drugs in COVID-19 patients. Associations are colour-coded (white when not calculated, grey when not significant, orange when significant only considering the CI 95%, red when significant also after Bonferroni multiple comparison).

**Table S2. Disproportionality analysis results**, showing RORs and p-values of events with at least one significant ROR before the correction. We colored in red those associations which passed the Bonferroni correction (α=0.000080775).
